# The Tricky Diagnosis of Nummular Headaches: Description of Two Cases and Literature Review

**DOI:** 10.7759/cureus.25043

**Published:** 2022-05-16

**Authors:** Haseeb Ikram, Haris Vakil, Kate Zipperer, Xiang Fang, Quratulanne Jan, Jamal Islam, Prashant Rai, Neeharika Thottempudi

**Affiliations:** 1 Family Medicine, The University of Texas Medical Branch at Galveston, Galveston, USA; 2 Neurology, The University of Texas Medical Branch at Galveston, Galveston, USA

**Keywords:** neuralgias, central sensitization, peripheral nociception, primary headaches, nummular headache

## Abstract

Nummular headaches are a rare and relatively newly characterized primary headache disorder. The epidemiology is largely unknown due to likely underdiagnosis and a small population of all headache patients in outpatient presentation. Though our understanding of nummular headaches continues to evolve, they remain a diagnostic challenge for physicians and the underlying pathophysiology is poorly understood. Hypotheses consider neuralgia stemming from epicranial tissues as well as undergoing observation of varying prevalence of autoimmune markers. Peripheral nociception versus central sensitization needs to be evaluated as well, with cases not having consistent direction. Selecting treatment options can be challenging due to limited efficacy, the vague nature of reported symptoms, the rarity of the diagnosis, and the range of presentations. Several treatment modalities have been utilized including non-steroidal anti-inflammatory drugs (NSAIDs), beta-blockers, botulinum toxin injection, transcutaneous nerve stimulation, or even simple reassurance. A case-by-case analysis must be undertaken to best develop treatment options for affected individuals as high-quality randomized quality trials for nummular headaches are very few. We detail two novel cases of patients presenting with nummular headaches that highlight the challenges and importance of making the diagnosis and weighing treatment options for improved levels of patient care, which is followed by a literature review.

## Introduction

Nummular headache is a primary headache disorder that is described as a well-circumscribed area of pain located in any region of the cranium. This subtype of primary headache is rare and often underdiagnosed, as they can present with different patterns of pain including continuous pain, relapsing-remitting pain with no pain between episodes, or chronic baseline pain with superimposed episodes of severe pain [1,2].

Nummular headaches were first characterized in 2002 in the now-classic paper by Pareja et al. [1]. By definition, a nummular headache cannot be attributed to any underlying lesion or structural defect, nor can it be defined by any other primary headache syndrome [3]. Pain episodes may be accompanied by allodynia, dysesthesia, paresthesia, and hypoesthesia [4]. Nummular headaches most often occur with greater frequency in the parietal region, but they can occur in other regions as well. Typically, unifocal nummular headaches may also have a multifocal presentation with the caveat that all symptomatic areas meet nummular headache criteria [1,5]. In a case series reported by the Mayo Clinic, around 56% of 16 patients had existing diagnoses of migraine headaches [6]. Patients with nummular headache and comorbid migraines noticeably differentiated between the two patterns of pain. Here we present two novel cases of nummular headaches and challenges encountered in diagnosis as well as treatment, in addition to a brief literature review.

## Case presentation

Case 1 

The patient was a 65-year-old Caucasian male with a past medical history significant for hypertension, depression, hepatitis C, and arthritis, who presented with headaches that began five months ago. He reported the onset of headaches following a nasal septoplasty with polypectomy. The headaches were located in the midline occipital region, and described as a sharp, stabbing pain without any radiation. According to the patient, he had never experienced a headache like this before. He denied any associated symptoms including nausea, vomiting, photophobia, phonophobia, or visual disturbance. 

The patient stated that his headaches had been constant since onset five months ago. They were quite debilitating and caused difficulty falling asleep, which led to profound fatigue. He tried multiple over-the-counter medications with no relief. He had also recently visited a chronic pain management physician and had been trialed on tizanidine, gabapentin, and Flexeril with no relief. The patient had also undergone two occipital nerve blocks. He reported that they helped improve his headache for two days, but returned with the same intensity as before. The patient had already had an MRI brain with contrast and magnetic resonance angiogram (MRA) of his head, which did not demonstrate any significant intracranial abnormalities. 

The patient’s neurological examination was normal; however, in the occipital region, he had a 6 x 7 cm ovoid area that crossed the midline and was exquisitely tender to touch. His laboratory findings and imaging were reviewed and he was diagnosed with a nummular headache. He was started on nortriptyline 10mg to be taken at night. 

On a three-month follow-up, the patient stated that he did not feel nortriptyline was helping his pain, so he discontinued it after only a week of use. Fatigue, headache, inability to carry out daily functions, and altered sleep habits continued. The patient was recommended to start topiramate 25 mg at night; however, the patient did not find relief and was lost to follow-up. 

Case 2 

The patient was a 66-year-old Asian American male who presented with a chief complaint of a new-onset headache that started one month ago. His past medical history is significant for hypertension, dyslipidemia, coronary artery disease, benign prostatic hypertrophy, C4-C7 mild spinal canal stenosis, and venous insufficiency of the lower extremities. The patient described the headache as a constant, dull pain in the right temporal region in a well-circumscribed area, roughly the size of a golf ball (4 x 4 cm). The patient also reported intermittent worsening of the constant headaches, during which time he experiences a sharp, debilitating pain that reaches 8/10 in intensity. This remains localized to the right temporal region without any radiation. He had been taking ibuprofen 400mg, which did help alleviate the pain. 

The headache continued for over a month and due to increasing concern, he saw a neurologist. His physical examination was unremarkable, and aside from the occasional tingling sensation in the right thumb, he did not have any weakness or altered sensation and had a full range of motion. After reviewing the medical history and physical exam, a diagnosis of nummular headache was made. The patient was prescribed topiramate 25mg for the headache. An MRI and MRA of the brain were ordered, which were normal. The patient continued topiramate without any pain relief and ultimately stopped taking the medication after three weeks. The patient eventually experienced an improvement in his symptoms over a three-month period without any further intervention. He was recommended to continue walking three-four days per week for at least 30 minutes without significant change or addition in medication.

## Discussion

The rarity of nummular headaches has been documented in a few hospital series with incidences of 6.4/100,000 a year, and 1.25% of all headache patients in an outpatient office [2,4,7]. The added complexity from low incidence coupled with equivocal treatment regimens makes for a challenging diagnosis and treatment. Pathogenesis is poorly understood as well, leading to further difficulties, and often patient and clinician frustration. 

Additional clinical research is necessary to distinguish peripheral nociception versus central sensitization. There are hypotheses that support localized neuralgia stemming from epicranial tissues such as terminal branches of a cutaneous nerve versus pain originating from terminal branches of the sensory nerve [8]. A higher prevalence of autoimmune markers was identified in a small study of 23 patients with primary nummular headache. Abnormal seromarkers included antinuclear factor antibodies and rheumatoid factor; however, the small sample size limits the conclusions that can be drawn [9]. Our cases did not present such variations in these seromarkers. 

The International Headache Society attempted to further clarify this definition. The third edition of the International Classification of Headache Disorders (ICHD-3) sought to define nummular headaches as pain that is located in a small, circumscribed area of the scalp and in the absence of any underlying structural lesion [8]. Notably, this definition of nummular headache heavily implies that it is a diagnosis of exclusion, supported by the full diagnostic criteria available for review (Table 1). Both of our cases met this requirement when evaluated. 

**Table 1:** Nummular headache ICHD-3 diagnostic criteria Adapted from: The International Classification of Headache Disorders (ICHD-3), 3rd edition [8] *If criterion D is met, the diagnosis is more specifically classified as ‘Probable Nummular Headache'.

Criterion	Finding
A	Continuous or intermittent head pain fulfilling criterion B
B	Felt exclusively in an area of the scalp with all of the following four characteristics: 1) Sharply contoured, 2) Fixed in size and shape, 3) Round or elliptical, 4) 1-6 cm in diameter
C	Not better accounted for by another ICHD-3 diagnosis
D	Not fulfilling ICHD-3 criteria for any other headache disorder*

Currently, there is no clearly defined treatment intervention in place. High-quality randomized controlled trials are unfortunately lacking given the underrecognized and novel nature of this diagnosis. There are several treatment modalities that have been utilized, with gabapentin being the most widely used with positive results [6]. Botulinum toxin injections have been used to treat nummular headaches with some success, though the toxin seems to wear off after three months of treatment with an unclear efficacy overall [10]. Beta-blockers have also been proposed as a possible therapeutic avenue [11]. NSAIDs (particularly indomethacin) have also been proposed as effective therapeutic options [12]. Other notable treatments include pregabalin, carbamazepine, neurotropin, and transcutaneous nerve stimulation [6,10-12]. Although some show promising results, no single therapy has given a complete resolution of symptoms [6]. Furthermore, therapeutic intervention is not always necessary and may only require simple reassurance [13].

A literature review of nummular headache cases by Dai et al. illustrates the diversity of the possible clinical presentations (Table 2), and the cases presented in this paper are no exception [14]. With respect to Case 1 in the current report, the patient presented with an occipital midline region of pain, which is a rare presentation in itself when compared to the more common regions of pain found in nummular headaches as seen in Table 2. The patient failed several therapeutic interventions with little to no relief. Eventually, the patient was amendable to taking a low dose of topiramate and managing the pain conservatively. It is unknown if reduction of pain was successfully achieved for the patient as he was lost to follow-up. In Case 2, the patient presented with a less common distribution of pain in the right temporal region, similar to what is seen in Table 2. After not finding marked improvement in pain with medication, this patient opted to undergo conservative management, which incidentally allowed symptom resolution. In both scenarios, medication intervention somewhat improved patient comfort but did not bring complete resolution of symptoms. The value of these two clinical cases is that they not only add to the small collection of cases already reported but also emphasize the importance of familiarity and the ability to distinguish nummular headaches from other primary headache syndromes. 

**Table 2:** Nummular headache clinical characteristics Source: Dai W, Yu S, Liang J, Zhang M [14] Reprinted by Permission of Sage Publications

Clinical Characteristic (n= number of cases with data)	Data Value
1.Gender(n=238)	
1a. Female	141 (59%)
1b. Male	97 (41%)
2.Age at Onset (n=196)	Mean at 44.4 years
3.Age at Reported(n=153)	Mean at 47.5 years
4. Headache duration (n=111)	Mean at 6.4 years
5. Location (n=191)	
5a. Parietal	85(44%)
5b. Occipital	42 (22%)
5c. Frontal	27 (14%)
5d. Temporal	24 (13%)
5e. Vertex	5 (3%)
5f. multi-region	8 (4%)
6. Side (n=207)	
6a. Left	116 (56%)
6b. Right	79 (38%)
6c. Middle	12 (6%)
7. Focal (n=206)	
7a. One	200 (93%)
7b. Bifocal	13 (6%)
7c. Multifocal	1 (0.5%)
8. Size (n=65)	Mean at 6.5 cm
9. Pain Quality (n=118)	
9a. Pressing	53 (45%)
9b. Stabbing	34 (29%)
9c. Burning	22 (19%)
9d. Throbbing	6 (5%)
9e. Other	3 (2%)

## Conclusions

Nummular headaches may very well co-exist with other types of headaches. An astute clinician must distinguish this diagnosis from secondary or local processes when clinically working up a patient’s headache. Navigating and identifying the various pain processes and the poorly differentiated pathophysiology of such a diagnosis adds to both the intrigue and characterization of our presented cases. While resolution of symptoms is typically found with conservative measures and reassurance, patients must be thoroughly evaluated on a case-by-case basis to uncover the true etiology of their pain and to reduce underdiagnosing of this novel, often missed, and underrecognized phenomenon. 
